# The Expression of a Xylanase Targeted to ER-Protein Bodies Provides a Simple Strategy to Produce Active Insoluble Enzyme Polymers in Tobacco Plants

**DOI:** 10.1371/journal.pone.0019474

**Published:** 2011-04-29

**Authors:** Immaculada Llop-Tous, Miriam Ortiz, Margarita Torrent, M. Dolors Ludevid

**Affiliations:** 1 Department of Molecular Genetics, Centre for Research in Agricultural Genomics (CRAG, Consortium CSIC-IRTA-UAB), Barcelona, Spain; 2 ERA Biotech, Barcelona, Spain; University of Crete, Greece

## Abstract

**Background:**

Xylanases deserve particular attention due to their potential application in the feed, pulp bleaching and paper industries. We have developed here an efficient system for the production of an active xylanase in tobacco plants fused to a proline-rich domain (Zera) of the maize storage protein γ-zein. Zera is a self-assembling domain able to form protein aggregates *in vivo* packed in newly formed endoplasmic reticulum-derived organelles known as protein bodies (PBs).

**Methodology/Principal Findings:**

Tobacco leaves were transiently transformed with a binary vector containing the Zera-xylanase coding region, which was optimized for plant expression, under the control of the 35S CaMV promoter. The fusion protein was efficiently expressed and stored in dense PBs, resulting in yields of up to 9% of total protein. Zera-xylanase was post-translationally modified with high-mannose-type glycans. Xylanase fused to Zera was biologically active not only when solubilized from PBs but also in its insoluble form. The resistance of insoluble Zera-xylanase to trypsin digestion demonstrated that the correct folding of xylanase in PBs was not impaired by Zera oligomerization. The activity of insoluble Zera-xylanase was enhanced when substrate accessibility was facilitated by physical treatments such as ultrasound. Moreover, we found that the thermostability of the enzyme was improved when Zera was fused to the C-terminus of xylanase.

**Conclusion/Significance:**

In the present work we have successfully produced an active insoluble aggregate of xylanase fused to Zera in plants. Zera-xylanase chimeric protein accumulates within ER-derived protein bodies as active aggregates that can easily be recovered by a simple density-based downstream process. The production of insoluble active Zera-xylanase protein in tobacco outlines the potential of Zera as a fusion partner for producing enzymes of biotechnological relevance. Zera-PBs could thus become efficient and low-cost bioreactors for industrial purposes.

## Introduction

Enzymes are currently used in several industrial products and processes and new areas of applications are constantly being added. Thanks to advances in biotechnology, novel technical enzyme production technologies offer great potential for many industries including the pulp and paper industry, feed and food industry, biofuel production and the textile industry [1]. Technical enzymes can be used as purified enzymes, partially purified enzymes or whole cells containing functional catalytic activities and are often obtained from a natural source or by recombinant expression in bacteria or yeast. There is also increasing interest in the use of plants as host expression systems for technical enzymes because their production can be easily scaled up with low production costs [2], [3].

Xylanases are bacterial enzymes that degrade xylans, breaking down hemicelluloses, which are one of the major components of plant cell walls. Xylanases are fast becoming a major group of industrial enzymes, with significant application in feed, pulp bleaching and paper industry [4]. Many different technologies have been developed to produce xylanases in plants using stable transformation methods. Bacterial xylanases for bio-bleaching and baking applications have been expressed in transgenic rice [5] barley [6] and *Arabidopsis*, targeted either to peroxisomes or chloroplasts, or at both organelles simultaneously [7]–[9]. Attempts to produce xylanases in the plant root exudates of transgenic tobacco plants [10] and in transgenic potato plants [11] have also been made. However, in all cases the level of enzyme activity was far below that required industrial application. Recently, approaches to producing industrial enzymes for the degradation of lignocellulosic biomass have focused on generating crop varieties that self-produce and store degrading enzymes. In this context, a bacterial xylanase was expressed in the endosperm of wheat seeds [12] but the wheat grains had a severe phenotype and plant fertility was affected. The expression of thermophilic enzymes that are inactive at ambient temperatures is an alternative approach to overcome the detrimental effects that were encountered when active cell-wall hydrolytic enzymes accumulated in the apoplast. Recently, two thermophilic bacterial xylanases targeted to the apoplast were expressed in *Arabidopsis thaliana*, and transgenic plants appeared phenotypically normal and fully fertile. Both xylanases were preserved in dried *Arabidopsis* stems with very low activities at 40°C but were active at 85°C and interestingly, extracts from dry stems showed a decrease in the molecular weight of xylans after heat treatment [13].

When using plants as a host for the expression of industrial enzymes three main factors are decisive in determining their commercial viability: i) high levels of expression ii) efficient downstream processing and iii) operational stability of enzymes. High expression levels have been obtained by using fusion protein technologies such as elastin-like polypeptides (ELPs) [14]–[16], hydrophobin [17], zeolin [18] and Zera, a proline-rich domain of the maize storage protein γ-zein [19]. All these fusion proteins when expressed in plants are encapsulated in ER-derived PBs. ELPs fusions proteins are recovered as soluble recombinant protein by a non-chromatographic separation method termed inverse transition cycling (ITC) [20], hydrophobin fusions by two-phase procedures [17] and Zera fusions by density-based methods [21]. The oleosin fusion expression system is another example of fusion technology that has been used to produce recombinant proteins on the surface of oil bodies, which can be separated by flotation from an aqueous extraction of seed biomass [22].

Cost-effective manufacturing of bulk enzymes requires a simple and rapid downstream processing of the bioactive enzyme preparations and a functional operational stability of the enzymes. In the last decade, immobilized and insoluble enzymes preparations have emerged as the optimum methodology to enhance operational performance of biocatalysts [23], [24]. In this regard, the ability of the proline-rich domain of γ-zein (Zera) to pack fusion proteins as insoluble aggregates within PBs [25] appears as a desirable attribute to produce industrial enzymes.

Our aim in the present study is to explore the applicability of Zera attributes to produce bioactive industrial enzymes in plants in their insoluble form. A xylanase mutant [26] of *Streptomyces olivaceoviridis* XYNB xylanase [27] was chosen as an industrial enzyme model. We show that xylanase fused to Zera (Zera-Xyl) is highly expressed in *Nicotiana benthamiana* transiently transformed leaves, accounting for up to 9% of the total soluble proteins. The Zera-Xyl fusion protein accumulates in dense protein bodies and the enzyme is post-translationally modified with ER maturation-type glycans. We demonstrate that, after its recovery simply by centrifugation, the insoluble polymer of Zera-Xyl is biologically active and shows stable activity for at least one month at room temperature.

## Results

### Transient expression of xylanase fused to a proline-rich domain (Zera) in tobacco leaves

**Figure 1:**
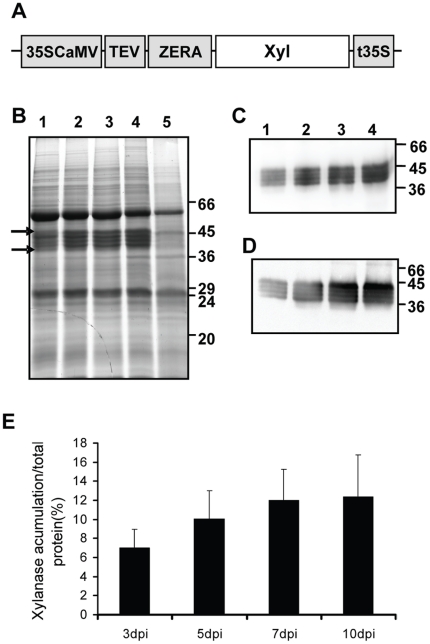
Transient expression of Zera-Xyl in *Nicotiana benthamiana* leaves. (A) Schematic representation of plant expression vector encoding for Zera xylanase fusion under the control of the enhanced cauliflower mosaic virus 35S promoter, a translational enhancer of tobacco etch virus (TEV) and the cauliflower mosaic virus 35S terminator (pCZera-Xyl). The Zera sequence includes the signal peptide and the first 112 amino acid coding region of γ-zein. (B) Coomassie-stained SDS-PAGE analysis of protein extracted from *N. benthamiana* leaves co-infiltrated with Zera-Xyl and the silencing suppressor HcPro. Leaf tissue was collected at 3 (lane 1), 5 (lane 2), 7 (lane 3) and 10 (lane 4) days post infiltration (dpi). Leaves agroinfiltrated only with HcPro (lane 5) were used as control. Gels were loaded with 20 µg of total protein and stained with Coomassie blue. Arrows indicate the molecular weight window of the multiple bands of Zera-xylanase. (C–D) Immunoblots blots were incubated with xylanase antibody (C) and with an antibody against Zera (R8 antibody) (D). In immunoblots, gels were loaded with 2.5 µg of total protein. (E) Levels of Zera-Xyl protein accumulation (%) relative to the total protein determined by densitometric analysis of Coomassie-blue-stained SDS-PAGE gels. Data were quantified with Multi Gauge v.3.0. The relative accumulation of Zera-Xyl was calculated on the basis of the total protein loaded in each lane corresponding to 3, 5, 7 and 10 dpi. The results represent the average of at least three independent protein extracts.

A codon-optimized XYNTB synthetic gene from *Streptomyces olivaceoviridis* [26] was fused at its N-terminus to the DNA sequence coding the signal peptide and proline-rich domain of γ-zein (Zera). The entire chimeric Zera-XYNTB gene (Zera-Xyl) was inserted into the plant binary expression vector pCambia 2300 under the control of the enhanced cauliflower mosaic virus (CaMV) 35S promoter, a TEV translational enhancer and the 35S terminator (Figure 1A, pCZera-Xyl construct). The construct was co-agroinfiltrated into *Nicotiana benthamiana* leaves together with a vector containing the coding sequence of the HcPro protein, a suppressor of silencing [28]. Transiently transformed *N. benthamiana* leaves were collected at 3, 5, 7 and 10 days post infiltration (dpi). Leaves transformed only with the silencing suppressor were used as controls. Coomassie staining of proteins resolved in electrophoretic gels showed several abundant bands ranging from 38 to 44 kD in protein extracts from leaves that had been transformed with the pCZera-Xyl construct (Figure 1B). The observed bands had a higher molecular mass than the 36 kD expected for the Zera-Xyl fusion protein. The identity of the fusion protein was established using polyclonal antibodies directed either against Zera (anti-R8 antibody) [19] or xylanase. Polyclonal antibodies against xylanase were raised in rabbits immunized with pure XYNTB expressed in *E. coli*. The western blot analysis of protein extracts from transformed leaves confirmed that the highly abundant bands corresponded to different forms of Zera-Xyl (Figure 1C and D, respectively). Because xylanase has three consensus N-glycosylation sites, the microheterogeneity detected is probably due to post-translational modification, as demonstrated later. The Zera-Xyl expression levels were determined by densitometry using an extract of total proteins of tobacco leaves transformed only with HcPro as reference. As shown in Figure 1E, the accumulation levels of Zera-Xyl isoforms, which were visualized by Coomassie staining as early as 3 dpi (Figure 1B), clearly increased up to 7 dpi and remained stable thereafter. According to the densitometry results, Zera-Xyl accounted for 12% of the total protein resolved in the SDS-PAGE (Figure 1B). However, quantification by densitometry only includes some of the total protein, and therefore we estimate that the fusion protein would account for approximately 9% of the total protein. This would correspond to 1.6 g of Zera-Xyl/Kg fresh weight or 1.08 g of xylanase/Kg fresh weight.

### Zera-xylanase protein accumulates in dense protein bodies

**Figure 2:**
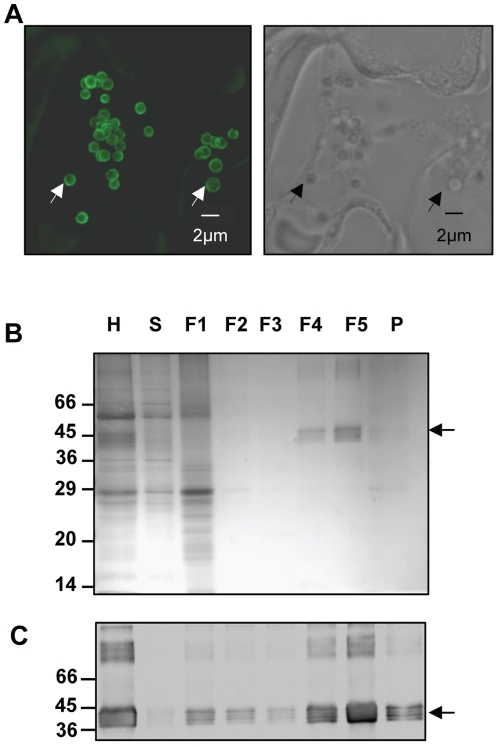
Zera-Xyl is stored in dense PBs. (A) Whole mount immunolocalization of Zera-Xyl in *N. benthamiana* leaves at 7 days after agroinfiltration. R8-antibody (1∶8000 dilutions) was used as the primary antibody and anti-rabbit IgG conjugated to Alexa Fluor 488 dye was used as the secondary antibody. In the confocal optical section on the left, white arrows indicate a couple of PBs immunolabelled in green. On the right, the black arrows in the transmission picture indicate the same PBs. (B–C) The density of Zera-Xyl-induced PBs was assessed by loading *N. benthamiana* homogenates onto a multi step Iodixanol density-based gradient (steps: 1.11, 1.17, 1.19, 1.21, 1.23, 1.25 g/cm^3^). Proteins in the homogenate (H), supernatant (S), fractions of increasing density (F1–F5) and pellet (P) were resolved in SDS-PAGE and stained with Coomassie blue (B) or analyzed by immunoblot (C). Immunoblot was probed with the Xyl antibody. Multiple immunoreactive forms of Zera-Xyl were recovered mainly in the F5 fraction (δ; 1.233 g/cm^3^) but also in the dense fraction F4 (1.214 g/cm^3^). Arrows indicate the recombinant Zera- Xyl fusion protein.

We then explored the subcellular localization of the Zera-Xyl fusion protein in tobacco leaf sections collected at 7 dpi. Immunolocalization was performed by whole mounting using anti R8 as the primary antibody and anti-rabbit IgG conjugated to Alexa Fluor 488 dye as the secondary antibody, with examination by confocal laser scanning microscopy. A characteristic of Zera fusion proteins is their ability to target and accumulate in PBs. As shown in Figure 2A, tobacco epidermal cells displayed numerous spherical organelles with diameters of 1–2 µm, similar in shape and size to the PBs previously described for other Zera fusions proteins [19], [25]. The bright green fluorescence was only observed in the periphery of the PB. This could be due to Zera domain polymerization [25], making the highly packed PBs inaccessible to the antibody.

The next step was to test whether the Zera-Xyl-accumulating organelles were dense, as has been described for a variety of Zera fusions [19], [25], [29]. To characterize the density of the PB-like structures accumulating Zera-Xyl, we analyzed Zera-Xyl-expressing leaf homogenates by subcellular fractionation using Iodixanol-based density step gradients (Optiprep) [21]. Equivalent amounts of collected fractions were analyzed by gel electrophoresis followed by Coomassie blue staining and immunoblot analysis using the anti-Xylanase antibody. As shown in Figure 2 (B,C), most of the Zera-Xyl protein was recovered in the dense 1.21–1.23 g/cm3 (F4) and 1.23–1.25 g/cm3 (F5) interfaces of the gradient, confirming the expected high density of the new induced organelles. The high molecular weight immunoreactive bands that were detected in the homogenate and in the dense fractions are likely to correspond to dimers and trimers of Zera-Xyl. We then investigated whether Zera-Xyl microheterogeneity, which was also observed in dense PBs, was the result of xylanase posttranslational modifications such as glycosylation.

### Recombinant Zera-xylanase present in protein bodies is glycosylated with high mannose-type glycans

**Figure 3:**
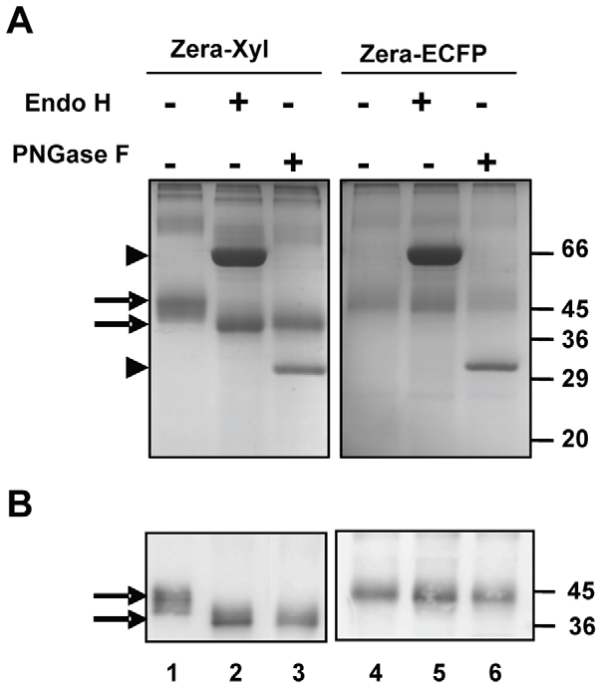
Glycosylation of Zera-Xyl. PB extracts prepared from Zera-Xyl transformed leaves were digested with Endo H and PNGase F as indicated and analyzed by gel electrophoresis stained with Coomassie blue. Non-digested Zera-Xyl was used as a reference (lane 1). The Zera-ECFP fusion was also digested with both glycosylases (lanes 4–6) as a control of non-glycosylated protein. Zera-Xyl bands were sensitive to both Endo H and PNGase F. The bands shifted to lower molecular weight bands (lanes 2 and 3 and arrows), whereas the unglycosylated Zera-ECFP protein was resistant to digestion (lanes 5 and 6). Lower panel: immunoblots using R8 antibody. Arrowheads indicate the electrophoretic bands of Endo H and PNGase F.

The XYNTB xylanase sequence contains three potential glycosylation sites but Zera has no consensus site for N-glycosylation. Therefore, the microheterogeneity of Zera-Xyl bands observed in the electrophoretic pattern (Figure 1) prompted us to investigate whether the xylanase was glycosylated when expressed as the Zera-Xyl fusion and accumulated in PBs. To determine the presence of glycans and their structure, PBs were recovered from Zera-Xyl-transformed leaves and were treated with Endo H and PNGase F glycosylases followed by SDS-PAGE and western blot analysis using the R8 antibody. As a non-glycosylated control, a preparation of PBs from Zera-ECFP-transformed leaves was treated with the two glycosylases in the same conditions. After treatment with Endo H and PNGase F, the broad band of Zera-Xyl showed a mobility shift as well as a reduction in the number of electrophoretic bands (Figure 3, lanes 1–3). Xylanase was sensitive to both Endo H and PNGase F. The smaller bands of about 36 kDa generated after enzymatic processing corresponded to the expected apparent molecular weight for the unglycosylated Zera-Xyl fusion. As expected, non-glycosylated Zera-ECFP was not sensitive to Endo H or PNGase F (Figure 3, lanes 4–6). The abundant bands of 60 kDa and 32 kDa observed in the Coomassie-blue-stained electrophoretic gels corresponded to Endo H and PNGase F respectively (Figure 3A arrowheads). These results reveal that the N-linked glycans of Zera-Xyl had high mannose structure, indicative of its localization in the ER [30], and also indicate that the addition of these glycans does not impair the self-assembly and polymerization of Zera that is required for PB formation.

### Xylanase activity is not compromised by the fusion with Zera

**Figure 4:**
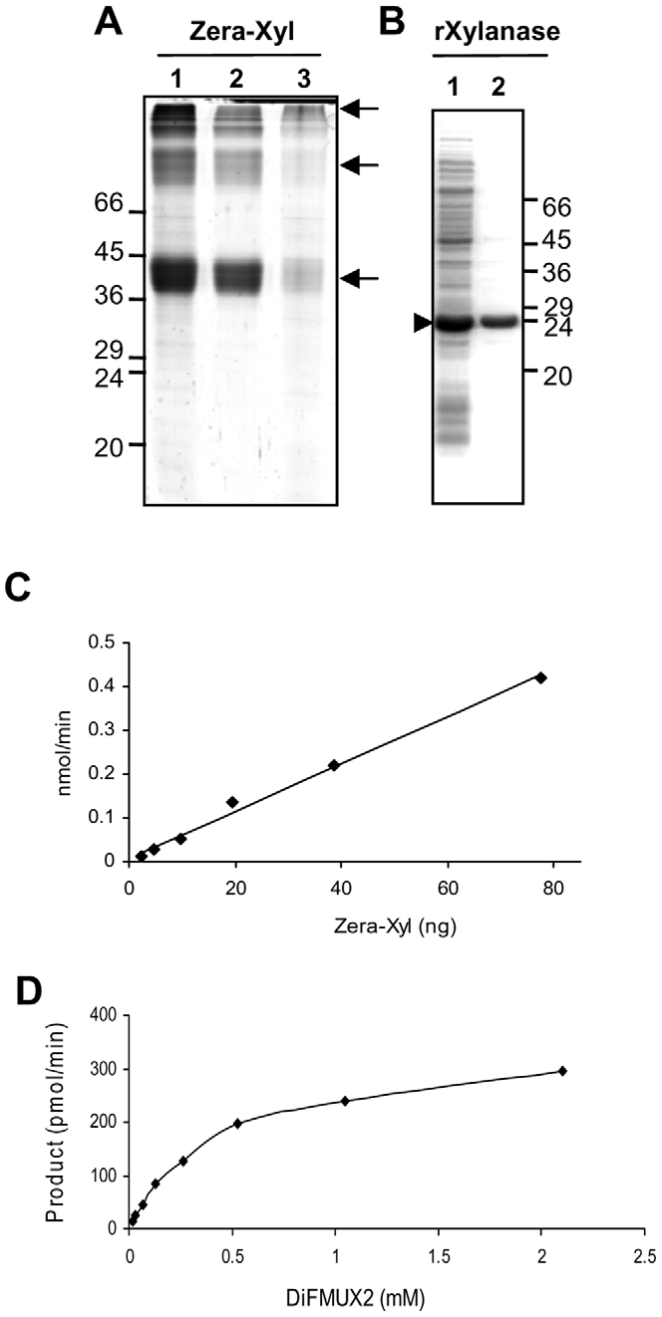
Activity of Zera-Xyl solubilized from PBs. (A) Gel electrophoresis analysis of Zera-Xyl fusion protein present in PBs isolated from transformed tobacco leaves (lane 1). Zera-Xyl solubilized from PBs in 0.02% SDS, 5 mM TCEP for 2 h at room temperature (lane 2). Fusion protein that remained insoluble after treatment (lane 3) Arrows indicate monomers and oligomers of Zera-Xyl. (B) rXylanase expressed in *E. coli* BL21 strain. Analysis of total proteins of cell extracts analyzed by gel electrophoresis after the induction of expression with 1 mM IPTG for 5 h at 37°C (lane 1, arrowhead). Recombinant xylanase purified by Ni+ affinity column (lane 2). (C) Increasing amounts of solubilized Zera-Xyl were assayed for enzymatic activity and the product measured at 2 min intervals. For each Zera-Xyl concentration, the increase in product between 2 and 4 min was used to calculate the rate of product/min. (D) Determination of Michaelis-Menten constant (K_m_) of Zera-Xyl solubilized from PBs. Graphical representation of velocity (pmol/min) as a function of the substrate concentration [S] (mM DiFMUX2).

As Zera fusions accumulate as large oligomers inside the PBs due to Zera oligomerization, we attempted to determine xylanase activity after solubilizing the Zera-Xyl polymers from PBs. The purity of the recombinant protein was assessed by Coomassie staining of proteins resolved in a SDS-PAGE gel (Figure 4A) and the protein concentration was evaluated using the EZQ Protein Quantitation Kit (Molecular Probes). As shown in Figure 4A, protein extraction from a PB suspension (lane 1) in aqueous buffer in the presence of 0.02% SDS and 5 mM of reducing agent (TCEP, Tris(2-carboxy-ethyl)phosphine hydrochloride) was sufficient to solubilize most of the Zera-Xyl present in the PBs (Figure 4A, lanes 2 and 3). After dialysis in 200 mM phosphate citrate pH 6 buffer, appropriate dilutions of soluble protein were used for the activity assay (EnzChek Ultra Xylanase Assay Kit, Molecular Probes). As a positive control, XYNTB xylanase bearing a His tag expressed in *E. coli* (rXylanase) and purified by IMAC columns (Figure 4B) was included in the assay. Additional controls were also included, such as the commercial xylanase from *Trichoderma viride* (Sigma) as a positive control and a Zera fused to human growth hormone (Zera-hGH) solubilized from PBs as a negative control.

Conversion of the substrate to the fluorescent product was measured at different time points every 2 minutes over a period of 30 minutes. The accumulation of fluorescent product using different amounts of Zera-Xyl revealed that Zera-Xyl solubilized from PBs was highly active. The rate of product formation per minute, determined according to the increase in fluorescence between the first two time points (2 to 4 min), was shown to have a linear correlation with the amount of enzyme used in the reaction over a range of 2.5 to 80 ng of Zera-Xyl (Figure 4C). The affinity of Zera-Xyl for the substrate was determined by measuring the rate of product formation at increasing concentrations of substrate (Figure 4D). The Michaelis-Menten constant (K_m_) of Zera-Xyl was 0.46 mM, which is lower than that reported for another xylanase using the same synthetic substrate [31]. The specific activity of Zera-Xyl solubilized from isolated PBs yields an average of 5.5 nmol/min/µg of fusion protein. This result was similar to the enzyme activity measured in rXylanase expressed in *E. coli* (10.2 nmol/min/µg protein) and commercial *Trichoderma viride* xylanase (2.3 nmol/min/µg of protein) using the same assay conditions. When expressed on the basis of fresh weight tobacco biomass, PBs isolated from 1 g of fresh tissue yielded enough soluble recombinant Zera-Xyl to produce an average of 3.3 µmol of product per min. As expected, no xylanase activity was detected in Zera-hGH used as negative control (not shown). These results indicated that xylanase activity was not essentially affected by the presence of Zera, suggesting that this proline-rich domain, when fused to the N-terminus of the enzyme, interferes little with the folding and the affinity for the substrate.

### Protein bodies as bioactive organelles

**Figure 5:**
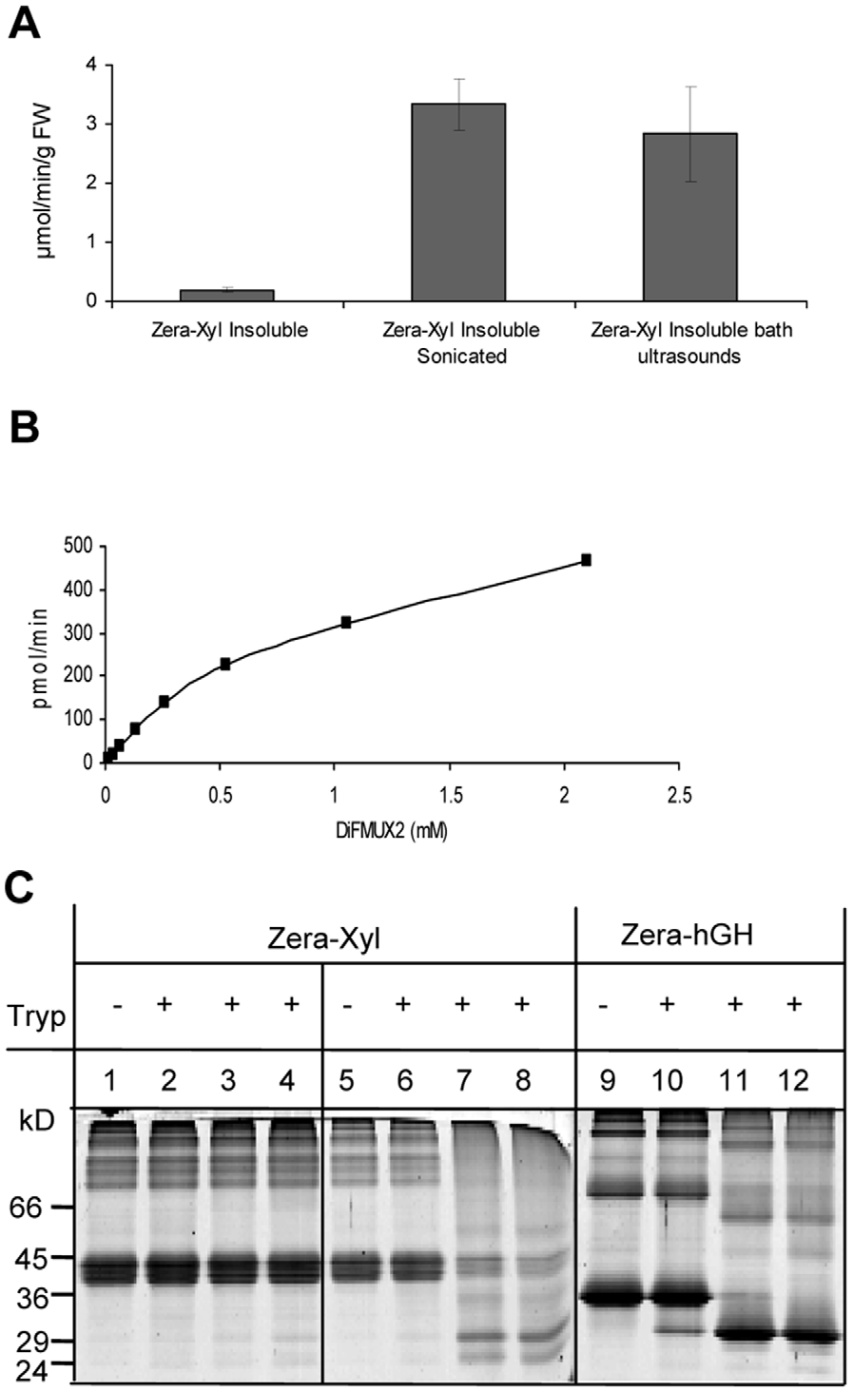
Activity of insoluble Zera-Xyl. (A) Insoluble Zera-Xyl was submitted to direct ultrasound or water-bath ultrasound before the enzymatic assay. Xylanase activity is referred to in µmol of product/min/g FW. (B) Determination of Michaelis-Menten constant (K_m_) of insoluble Zera-Xyl from PBs. Graphical representation of velocity (pmol/min) as a function of the substrate concentration [S] (mM DiFMUX2). (C) Zera-Xyl resistance to trypsin digestion. Zera-Xyl suspension was treated with trypsin at a trypsin∶Zera-Xyl ratio of 1∶200 (w∶w). Lane 2 represents time 0 of digestion. Two controls were included in the trypsin reactions: a Zera-Xyl suspension thermally denatured at 90°C for 20 min prior to trypsin treatment (lanes 5–8), and a suspension of Zera-hGH PBs isolated from Zera-hGH agroinfiltrated leaves (lanes 9–12). Trypsin reactions were carried out at 37°C for 0 h (lanes 2, 6 and 10), 1 h (lanes 3, 7 and 11) and 2 h (lanes 3, 7 and 12). Protein profiles were analyzed by gel electrophoresis stained with Coomassie blue.

With regards large-scale downstream processing of the protein, solubilization of the fusion protein could be an inefficient step. Therefore, we investigated whether Zera-Xyl was active in the oligomerized-insoluble form accumulated in PBs. To this end, PBs isolated from transformed leaves (see experimental procedures) were used directly for xylanase activity assays, avoiding prior solubilization of the recombinant protein. After washing of PBs, the large oligomers of Zera-Xyl recovered by centrifugation were maintained in a homogeneous suspension in the activity buffer by continuous shaking of the sample. Dilutions of the insoluble Zera-Xyl suspension were submitted to a xylanase activity assay by adding the synthetic substrate and measuring the fluorescent product. Interestingly, Zera-Xyl large oligomers (named insoluble Zera-Xyl) were also active, generating around 0.26 µmol of product/min/g FW (Figure 5A). However, the activity recovered per gram of fresh tissue was rather much lower than that obtained with solubilized Zera-Xyl. The reduced xylanase activity obtained from insoluble Zera-Xyl compared with that of solubilized Zera-Xyl could be due to the oligomerization process of Zera. Two main factors could explain the reduction in xylanase activity: i) the low accessibility of the substrate to the enzyme and ii) disruptions in the structure of the enzyme that would result in the presence of inactive forms of xylanase in PBs.

To explore these possibilities, we first considered the improvement of substrate accessibility to the enzyme by physical treatments such as directly applied sonication and immersion in ultrasound-bath. The application of both physical treatments to the polymer of Zera-Xyl resulted in a dramatic increase in xylanase activity (Figure 5A), reaching activity levels per gram of fresh tissue similar to those obtained with the solubilized enzyme. The Michaelis-Menten constant (K_m_) of insoluble Zera-Xyl was 1.19 mM (Figure 5B), substantially higher than that determined in soluble Zera-Xyl. Sonication treatment improved substrate accessibility but did not cause the Zera-Xyl polymer solubilization, as deduced from the absence of xylanase activity in the supernatants after centrifugation of the sonicated Zera-Xyl suspension.

Next, we investigated whether the insoluble Zera fusion contained a mixture of folded active and unfolded inactive forms of xylanase. To answer this question, we took advantage of the fact that the Zera peptide is resistant to trypsin proteolysis and that the activity of native xylanase XYNB is 100% resistant to trypsin digestion [32]. A suspension of insoluble Zera-Xyl prepared from PBs was digested with trypsin at pH 8 (Figure 5C, lanes 1–4). As controls, two additional digestions were carried out: the digestion of a suspension of Zera-Xyl previously treated at 90°C which denatured xylanase (Figure 5C, lanes 5–8) and the digestion of a suspension containing Zera fused to a trypsin sensitive protein such as human growth hormone (hGH) [33] (Figure 5C, lanes 9–12). As shown in Figure 5C (lanes 1–4) the protein profile of Zera-Xyl was not affected after 2 h of trypsin digestion (Figure 5C, lane 4), indicating that xylanase was resistant to the protease. In contrast, protein digestion was observed after 1 h of trypsin treatment in denatured Zera-Xyl (lane 7) and in Zera-hGH (lane 11). Moreover, the insoluble enzyme fully retained its original activity after 2 h digestion at 37°C (Table 1). However, prior treatment of Zera-Xyl at 90°C for 20 minutes resulted in the complete loss of activity (Table 1), indicative of effective denaturation of the enzyme, which was accompanied by xylanase susceptibility to proteolytic digestion. Altogether, these results indicated that the folding of xylanase was not disrupted by the polymerization of Zera. Therefore, Zera-Xyl-induced PBs essentially contained biologically active xylanase protein.

**Table 1:** Resistance of Zera-Xyl to trypsin digestion.

			Relative xylanase activity
**Zera-Xyl undigested**			**100**
**Zera-Xyl Trypsin digested**	Non-Denaturing Treatment	0 h	**106±14**
		2 h	**98±10**
	Denaturing Treatment	0 h	**0.3**
		2 h	**0.3**

A suspension of Zera-Xyl was digested with trypsin for 2 h at a trypsin∶Zera-Xyl ratio of 1∶200 (w∶w). Prior digestion, Zera-Xyl was either non-denatured or denatured by treating at 90°C. Xylanase activity was measured prior and after trypsin digestion.

### Stability of Zera xylanase activity

**Figure 6:**
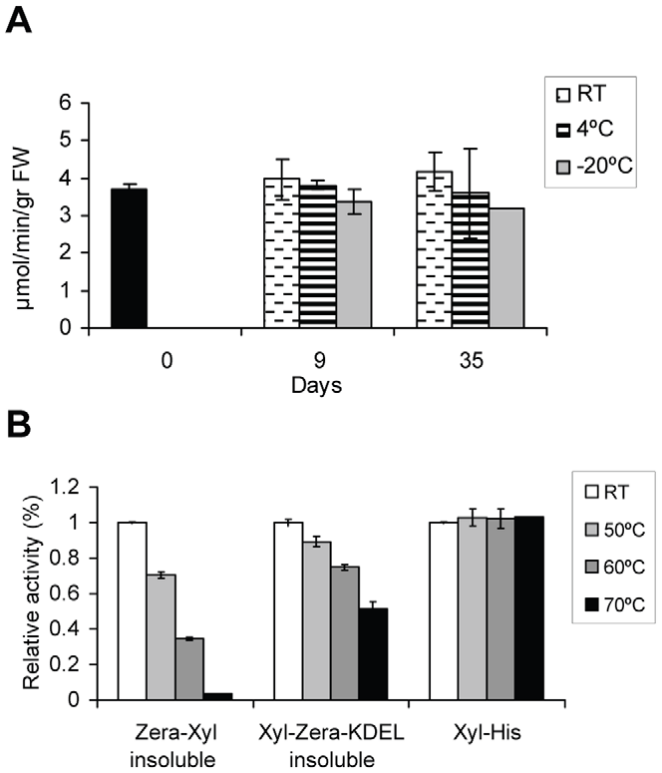
Operational stability and thermostability of insoluble Zera-Xylanase. (A) Measurement of Zera-Xyl activity at day 0, after 9 days and after 35 days at the temperatures indicated. (B) Thermostability of insoluble Zera-Xyl and insoluble Xyl-Zera-KDEL expressed in tobacco leaves and purified soluble rXylanase expressed in *E. coli*. Relative residual activity after incubation for 20 min at the temperatures indicated before the activity assay. Activities at RT were designated as 100% activity. Error bars represent the relative standard deviations of three independent replicates.

We also explored the stability of Zera-Xyl aggregates during storage at different temperatures. To this end, a freshly prepared Zera-Xyl suspension was divided into several aliquots that were kept either at RT, 4°C or −20°C for 9 or 35 days (Figure 6A). Our results indicate that the activity of insoluble Zera-Xyl is preserved for at least 35 days at both room temperature and at 4°C. Although a decrease in activity was observed at freezing temperatures, the enzyme retained more than the 80% of its activity at −20°C.

A bottleneck in the use of xylanases for industrial applications is the resistance of the enzyme to high temperatures [4]. Since the N-terminus of xylanase is involved in thermostability [26], two Zera protein fusions were used to study the effect of high temperatures on the activity of xylanase fused to Zera: Zera-Xyl and Xylanse-Zera-KDEL (Xyl-Zera-KDEL). This second construct allowed the enzyme to maintain the N-terminus free of fusion with Zera and, moreover, took advantage of the fact that PBs were also induced when Xyl-Zera-KDEL was expressed in tobacco leaves. Both the level of protein accumulation and efficiency of PB recovery of Xyl-Zera-KDEL were similar to those detected in Zera-Xyl (not shown). Insoluble Zera-Xyl and Xyl-Zera-KDEL preparations were incubated for 20 min at 50°C, 60°C and 70°C before the activity assay (Figure 6B). rXylanase expressed in *E. coli* was included as a positive control. In order to detect variations in activity, activity at room temperature was designed as 100% of activity. Under standard assay conditions, Zera C-terminal fusions improved thermostability, Zera-Xyl lost 70% of its activity after treatment at 60°C whereas the activity of Xyl-Zera-KDEL was reduced by only 25%. Moreover, Xyl-Zera-KDEL retained 50% of its activity after treatment at 70°C but Zera-Xyl was not resistant in these conditions. In the absence of Zera, rXylanase expressed in *E. coli* was fully active after 20 min at either 60°C or 70°C (Figure 6B) but lost its activity at 90°C (not shown).

## Discussion

In this study, we show that a fusion strategy can be very efficient at producing industrial enzymes in plants. A bacterial xylanase, used as a model enzyme, was fused to a proline-rich domain of γ-zein, which has the capacity to self-assemble *in vitro* [34] and in transgenic plants [35]. When this proline-rich domain (named Zera) was fused to target proteins it was also able to self assemble and to form large polymers inside the ER-derived PBs. The recombinant protein fusions remained encapsulated and surrounded by ER membrane. Subsequently, protein targeting to this subcellular organelle provided protein stability that resulted in high levels of accumulation of recombinant protein [19]. It has previously been shown that, as occurs in natural PBs from maize seeds [36], Zera-induced PBs in tobacco leaves can easily be recovered by density [21].

Our aim in using Zera fusions in this study was to produce active xylanase in plant PBs. Zera-Xyl constitutively expressed in *Nicotiana benthamiana* leaves increased the production of the enzyme. The Zera-Xyl fusion protein in total protein extracts was clearly detected until 10 dpi in electrophoretic gels stained with Coomassie blue. We estimated that the amount of Zera-Xyl in transformed leaves collected at 7 dpi represents up to 9% of the total protein. There is evidence that transient transformation of tobacco leaves is a very efficient system for producing rapid and scalable high rates of recombinant proteins [37]. The stability of the mRNA of Zera fusions determines, however, the period of time over which the protein is translated, oligomerized and accumulated in PBs. When using the agroinfiltration technique in transient transformations in tobacco, the mRNA of Zera fusions usually decayed after 7 dpi [25]. We have evidence that Zera fusion proteins are essentially stably accumulated in tobacco plants [19], and therefore levels of Zera-Xyl protein in transiently transformed tobacco leaves are likely to be regulated mainly by the stability of its RNA and enzyme accumulation in PBs.

As previously demonstrated for Zera protein fusions expressed in plants and in non-plant hosts [19], the storage of Zera-Xyl in dense ER-derived PBs favors its easy recovery by low-speed centrifugation. Targeting of recombinant proteins to ER-derived PBs has also been described using fusions of the target protein to ELP [16] and hydrophobins [17] In these two cases, however, the PBs formed were not dense, and therefore the recombinant protein had to be recovered by other means such as inverse transition cycling (ITC) and two-phase procedures. In order to improve recombinant xylanase production, several studies have considered xylanase expression targeted to different organelles. To date, the highest xylanase accumulation in leaves has been obtained by expressing xylanase targeted to the chloroplast [7]. We show here that Zera-Xyl fusion emerges as a powerful tool to produce this enzyme.

In our study, Zera-Xyl was not expressed as in a unique protein form. To our knowledge, the Zera domain is not glycosylated but the XYNTB xylanase amino acid sequence has three potential N-glycosylation sites that were glycosylated when expressed in *Pichia pastoris* [27]. A bacterial xylanase, similar to that used in this study, was also glycosylated when expressed in the apoplast of transgenic potato plants [11]. Zera-Xyl digestions with Endo H and PGNase F indicate that xylanase was glycosylated in PBs with high mannose-type glycans, consistent with its residence in the ER. The observed microheterogeneity of Zera-Xyl bands (ranging from 38 kDa to 44 kDa) suggests that after the transfer of the oligosaccharide precursor Glc3Man9GlcNAc2 onto the nascent Zera-Xyl, the kinetics of oligomerization of Zera inside the lumen of the ER [25] could determine the accessibility of glucosidases and glycosyltransferases [38] to the enzyme and, as result, the xylanase in PBs is trimmed with different N-glycan structures. Although complete N-glycans maturation would be impaired during the insolubilization of the enzyme within the ER, it should be noted that we did not observe degradation of the glycosylated fusion protein. It is likely that glycosylated Zera fusion proteins escaped the misfolded protein degradation pathway [39] avoiding binding to the calnexin/calreticulin folding cycle, or by saturation of the Hrd1/Yos9p-dependent ERAD pathway [40] due to the early formation of oligomers.

Zera-Xyl was stored in PBs as an insoluble protein, and hence we had two options for confirming its biological activity from a PB preparation: i) in solubilized Zera-Xyl samples or ii) in a suspension of insoluble Zera-Xyl directly prepared from PBs. The specific activity of solubilized Zera-Xyl yields an average of 5.5 nmol/min/µg of fusion protein, which is similar to that measured for the same xylanase expressed in *E. coli*. A more relevant finding was that insoluble Zera-Xyl was also active and that its activity was stable for at least a month at room temperature, indicating that, after a low-speed centrifugation of the biomass, PB preparations can be stored without requiring immediate processing. During the short downstream processing of tobacco biomass, we demonstrated that it is necessary to introduce an additional step consisting in ultrasound treatment. The accessibility of the substrate after this physical treatment restored the activity found in solubilized Zera-xylanase per gram of fresh tissue.

Thermostability is an important feature of xylanases for their industrial applications [4], [41] and is defined as the capacity of an enzyme to remain stable and active after being submitted to high temperatures (i.e. 70°C). The *Streptomyces olivaceoviridis* xylanase gene used in this work had been previously improved for its thermostability [27] by the substitution of the first 33 amino acids of the N-terminus with the 31 amino acids of a xylanase gene of *Termomonospora fusca* [26], [42]. We show that the fusion of Zera at the N-terminus of the enzyme leads to loss of thermostability relative to the fusion-free xylanase. Thus, rXylanase expressed in *E. coli* fully retained its activity at 60°C whereas Zera-Xyl lost 70%. Fusions of xylanase with Zera at the C-terminus containing a KDEL extension partially resolved the loss of activity. The recombinant Xyl-Zera-KDEL protein that also accumulated in PBs as an insoluble protein retained 75% of its activity after treatment at 60°C. Although this is a significant improvement, engineering of a modified xylanase should be carried out to optimize the use of Zera fusions in industrial applications. Recently, a systematic mutagenesis study on the N-terminus of the XYNB xylanase of *Streptomyces olicaceoviridis* showed that rather than a N-terminal replacement, substitution of a few critical residues dramatically improves xylanase thermostability [43].

XYNB xylanase is resistant to trypsin and pepsin digestion [27]. Trypsin digestion of Zera-Xyl allowed us to determine whether xylanase fused to Zera was active and, consequently properly folded. Both Zera-Xyl protein and xylanase activity were resistant to trypsin digestion. As the Zera domain has no trypsin-cleavable sites, the results suggest that if xylanase is not digested it should be properly folded when it is fused to Zera and accumulated in tobacco PBs. Thus, the self-polymerization process of Zera by hydrophobic interactions and disulfide bond cross-linking does not interfere during the folding process of the enzyme. The 3D structure of G11 xylanases including XYNTB xylanase, comprises two twisted β-sheets forming a so-called jellyroll, one α-helix and a region containing two glutamic acid residues between the two β-sheets that build the active and the substrate binding sites [44]. Although Zera-Xyl seemed to be properly folded, the affinity of insoluble Zera-Xyl for the substrate was lower than that measured in solubilized Zera-Xyl. In the light of this result we hypothesize that the protein concentration in the large Zera-Xyl oligomers impairs the conformational fluctuations, namely “breathing” [45], which would be needed to accommodate the substrate in the optimum environment. This result was not unexpected because in insoluble enzymes the diffusion rates of substrates through the aggregate may be limited [46]. We are currently engineering more swollen Zera-Xyl polymers to improve enzyme efficiency.

### Conclusion

To conclude, the present work outlines the production of a xylanase fused with a proline-rich domain (Zera) that is able to form large insoluble oligomers *in planta*. The production of fusion protein reached 1.6 g of Zera xylanase/kg, which corresponds to 1.08 g of xylanase/Kg of biomass. Xylanase in fusion was glycosylated, trimmed with N-type glycans and accumulated within dense ER-derived PBs in tobacco cells. The polymer of Zera-Xyl can easily be recovered by density means in a simple downstream process. Our successful production of insoluble active Zera-xylanase in tobacco demonstrated the feasibility of this approach for producing multiple enzymes of biotechnological relevance inside PBs. Zera-PBs could thus become efficient and low-cost bioreactors for industrial purposes.

## Materials and Methods

### Construction of plant expression vectors

A codon-optimized version of *Streptomyces olivaceoviridis* xylanase XYNTB gene (GenBank accession number: DQ465452) preceded by an enterokinase (EK) cleavage site was synthesized for tobacco expression. *NcoI* and *BamHI* restriction sites in the synthetic gene were used to replace ECFP in pUC18Zera-ECFP [19] with the xylanase sequence (obtaining pUC18Zera-Xyl). The translational fusion construct Zera-Xyl was inserted into the binary vector pC2300 containing the enhanced 35S promoter, the tobacco etch virus (TEV) translation enhancer and the 3′ polyadenylation sequences from the cauliflower mosaic virus (CaMV) yielding vector pCZera-Xyl.

The Xylanase-Zera-KDEL construct was obtained by amplifying SPg-Xylanase and Zera-KDEL coding sequences independently by PCR. The xylanase fragment was amplified from pUC18Zera-Xyl by PCR using 5′ overlapping primers, which introduced the γ-zein signal peptide coding sequence (SPg) preceded by a *Sal*I restriction site, and a 3′ reverse primer to introduce an *Asc*I restriction site. The resulting SPg-Xylanase fragment was inserted in the binary vector pCZera-ECFP [25] by *Sal*I*/Asc*I digestion to obtain the intermediate pCSPXyl-ECFP. The Zera-KDEL moiety was amplified from pUC18Zera-ECFP [19] using two overlapping 5′ primers designed to amplify the Zera sequence (lacking the signal peptide) preceded by an *Asc*I restriction site, a sequence coding for Lumiotag (CCPGCC) and a sequence coding for five glycine residues (linker). The 3′ reverse primer introduced the KDEL and a *Bam*HI restriction site. The Zera-KDEL fragment was introduced into the intermediate pCSPXyl-ECFP by *Asc*I-*Bam*HI to obtain pCSPXyl-Zera-KDEL. The pCZera-hGH construct used as control was prepared as described earlier [47].

To produce xylanase in *E. coli*, the xylanase coding sequence flanked by *Nco*I and *Xho*I restriction sites was amplified by PCR from pCZera-Xyl and inserted into the *Nco*I/*Xho*I digested pET28b vector. The resulting vector, named pETXyl, contained the XYNTB xylanase sequence fused in frame to the 5′ His-tag coding sequence of the vector.

### Plant material and transient expression system

Leaves of 4–6-week-old *Nicotiana benthamiana* plants were agroinfiltrated using the syringe method [48] with a culture of *Agrobacterium tumefaciens* strain EHA 105 transformed with pCZera-Xyl, pCSPXyl-Zera-KDEL, pCZera-ECFP [25] and pCZera-hGH [47]. All transiently transformed leaves were co-infiltrated in a 1∶1 ratio with an *Agrobacterium* culture transformed with a pCambia 2300 vector containing the HC-Pro silencing suppressor [28].

### *Escherichia coli* xylanase expression and anti-xylanase antibody

*Escherichia coli* BL21 cells transformed with pETXyl were grown to 0.5 OD and xylanase expression was induced with 1 mM IPTG for 5 h at 37°C. The cells were lysed by sonication and the soluble recombinant protein (rXylanase) was purified by Ni2+ affinity chromatography (Chelating Sepharose Fast Flow, GE Healthcare). The recombinant protein was eluted with 200 mM imidazole. Purified rXylanase was dialyzed against 200 mM phosphate-citrate buffer pH 6 for the activity assay and quantified using the EZQ Protein Quantitation Kit (Invitrogen, Molecular Probes). The polyclonal antibody against the enzyme was raised in rabbits injected with the purified rXylanase. In immunoblots, anti-xylanase antiserum was used at 1∶2000 dilutions.

### Protein extraction and immunoblot analysis

Proteins were extracted from 20 mg of agroinfiltrated leaf tissue in 250 µl 50 mM Tris-HCl buffer pH 8, 200 mM NaCl, 1% SDS, 2% β-mercaptoethanol and protease inhibitors for 1 h at RT. The resulting extracts were centrifuged at 10000×g for 10 min at RT. Protein extraction from untransformed leaves was also performed under similar conditions. Protein extracts were quantified using the Bradford reagent and analyzed by SDS-PAGE (12.5% polyacrylamide). Proteins were detected by both Coomassie blue staining and by immunoblot using either anti-R8 antibody [19] or anti-xylanase antibody.

### Endoglycosidase H and N-glycosidase F digestions

The Zera-Xyl protein obtained from PBs isolated by subcellular fractionation was deglycosylated with Endo H (New England Biolabs) or PNGase F (New England Biolabs) for 2 h at 37°C according to the manufacturer's instructions. As the unglycosylated protein control, a Zera-ECFP protein suspension was also digested with Endo H or PNGase F. Zera-Xyl and Zera-ECFP protein samples treated in the same conditions but with no enzyme were used as controls. Samples were analyzed by SDS-PAGE and immunoblot using the anti-R8 antibody.

### Immunocytochemistry and confocal microscopy

Whole mounting of wild type and agroinfiltrated leaves was carried out as described previously [49]. Pieces of infiltrated leaves were fixed in 4% paraformaldehyde with 0.1% Triton X-100 and permeabilized in Driselase (Sigma) and IGEPAL CA-630 (Sigma) plus 10% DMSO. For immunocytochemistry, the samples were pre-blocked in 2% BSA and incubated with anti-R8 antibody labelled with an anti-rabbit IgG conjugated to Alexa Fluor 488 dye (Molecular Probes). Green fluorescent images were collected at a wavelength of 488 nm excitation using an emission window set at 495 to 535 nm with a confocal laser scanning microscope (Olympus FV1000).

### Subcellular fractionation and protein body preparation

Subcellular fractionation in multi-step Iodixanol (Optiprep, Sigma) density gradients was carried out essentially as described previously [21].

Protein body preparation was performed from *N. benthamiana* leaves transiently transformed with Zera-Xyl fusion constructs. Four grams of leaf biomass were homogenized in 4 ml of cold PBP3 buffer (100 mM Tris-HCL pH 8, 50 mM KCl, 6 mM MgCl_2_, 10 mM EDTA, 0.4 M NaCl and protease inhibitors) at room temperature and filtered through two layers of Miracloth (22–24 µm, Calbiochem). The filtered homogenate was centrifuged at 1500×g for 10 min at 4°C. The pellet containing PBs was washed three times with 1% Triton and 0.5 M NaCl. The supernatants were discarded. Washed PBs containing insoluble Zera-Xyl fusion protein, were brought into suspension with 1 ml of the appropriate enzyme activity buffer or resuspended in Laemmli sample buffer for protein quantification analysis using the EZQ Protein Quantitation kit (Invitrogen, Molecular Probes).

### Xylanase activity assay

Xylanase activity was measured using an EnzChek xylanase assay kit (Molecular Probes) according to the manufacturer's protocol. Briefly, washed Zera-Xyl and Xyl-Zera-KDEL PBs were used directly or after sonication. Sonication was carried out in a digital Sonifier (Branson Model 450) at 2 sec/pulse and 10% amplitude for 30 sec on ice, or in ultrasound water bath (Selecta) for 20 min at 4°C. Suspensions were stirred and serially diluted in 200 mM phosphate citrate buffer pH 6. Fifteen µl of each diluted suspension were transferred to a 96-well ELISA plate and mixed with 50 µl of 52.5 µM synthetic substrate DiFMUX_2_. Fluorescence was measured on a *Synergy 2* Microplate reader (*BioTek* Instruments, Inc., Winooski, VT) every 2 min for 30 min at excitation/emission wavelengths of 360/40 nm and 460/40 nm, respectively, acquired with Gene 5 software. Commercial xylanase (*Tricoderma viride*, Sigma) and a washed PBs of Zera-hGH were used as positive and negative controls, respectively.

To determine the activity of solubilized Zera-Xyl, freshly prepared and washed PBs containing Zera-Xyl polymers were solubilized in 200 mM phosphate citrate buffer pH 6, 0.02% SDS, 5 mM of Tris (2-carboxy-ethyl) phosphine hydrochloride (TCEP, Sigma) and a cocktail of plant protease inhibitors (Sigma), with shaking for 2 h at room temperature. After centrifugation at 10000×g for 10 min, the supernatant was collected and dialyzed against 200 mM phosphate citrate buffer pH 6. Protein quantification and the purity of dialyzed samples were evaluated prior to preparation of serial dilutions in 200 mM phosphate citrate pH 6 for activity measurements. rXylanase and commercial xylanase from *Trichoderma viride* (Sigma) were used as positive controls. For determination of the Michaelis-Menten constant (Km), approximately 3 ng of soluble or insoluble but sonicated Zera-Xyl protein were incubated with substrate concentrations ranging from 16 µM to 2100 µM. The Michaelis-Menten constant (Km) was determined using Grafit32 software.

To evaluate storage stability, three replicates of protein suspensions were sonicated and processed immediately to determine activity at time 0 or were kept at different temperatures (RT, 4°C or −20°C) for 9 and 35 days. After the storage period, activity was measured as described above. To evaluate thermostability, three replicates of sonicated suspensions were maintained at RT, 50°C, 60°C or 70°C for 20 min before being transferred to ice and analyzed for activity.

### Trypsin digestions

The sensitivity of Zera-Xyl to tryptic digestion was determined as follows. Insoluble fusion protein from washed PBs was brought into suspension with 10 mM Tris-ClH pH 8. After sonication (2 sec pulse at 10% for a period of 30 sec), aliquots of this suspension were withdrawn and maintained on ice for subsequent analysis of proteins and xylanase activity in the absence of trypsin. The remaining Zera-Xyl suspension was incubated with trypsin (T1426 Sigma) at a ratio of 1∶200 (w∶w) for trypsin∶Zera-Xylanase, at 37°C. Digestion by trypsin was terminated at 0 h, 1 h or 2 h by the addition of 0.35 mg/ml of Soybean Trypsin Inhibitor (T9003 Sigma). After each incubation period, aliquots were taken for the analysis of proteins and xylanase activity. Zera-Xyl denatured at 90°C for 20 min was used as the unfolded xylanase control. Proteins were analyzed in 12% polyacrylamide SDS-PAGE gels. For measurement of xylanase activity, samples were centrifuged to recover protein aggregates and suspended in xylanase activity buffer (200 mM phosphate citrate, pH 6).
